# Supplementary material for: Periodic temperature changes drive the proliferation of self-replicating RNAs in vesicle populations
Source: Nat Commun. 2023 Mar 3;14:1222. doi: 10.1038/s41467-023-36940-z (PMC9984477; doi:10.1038/s41467-023-36940-z)
Supplement: Supplementary file 1 — Supplementary Information [file 41467_2023_36940_MOESM1_ESM.pdf]

Supplementary Information for

Periodic temperature changes drive the proliferation of self-replicating RNAs in vesicle populations

Elia Salibi[1§], Benedikt Peter[2§], Petra Schwille[2*], Hannes Mutschler[1*].

[1] TU Dortmund University, Department of Chemistry and Chemical Biology, Otto-Hahn-Str. 4a, 44227 Dortmund, Germany

[2] Max Planck Institute of Biochemistry, Department of Cellular and Molecular Biophysics, Am Klopferspitz 18, 82152 Martinsried, Germany

[§]These authors contributed equally

*Co-corresponding authors: schwille@biochem.mpg.de, hannes.mutschler@tu-dortmund.de

Table of Contents

Table of Contents 1

Materials 2

Supplementary Tables 3

Supplementary Figures 4

Supplementary Figure 1: Secondary structures of RNA systems used in the study. 4

Supplementary Figure 2: Time course of hammerhead activity *in vitro* supplemented with sucrose. 5

Supplementary Figure 3: Hammerhead activity in GUVs. 6

Supplementary Figure 4: Hammerhead activity in GUVs in the absence of freeze-thaw cycling. 7

Supplementary Figure 5: Effect of $MgCl_2$ on emulsion transfer GUV preparation. 8

Supplementary Figure 6: *In vitro* characterization of the enzymatically synthesized TAMRA-B-BHQ2 (FQ-B) substrate. 9

Supplementary Figure 7: Confocal microscopy analysis of freeze-thaw driven activity of a split R3C reporter ligase system. 10

Supplementary Figure 8: Membrane interactions of encapsulated RNA. 11

Supplementary Figure 9: Fluorescence intensity inside GUVs decreases upon freezing and thawing. 12

Supplementary Figure 10: Gel electrophoresis of RNA ligation by R3C ligase ribozyme F1 *in vitro*. 13

Supplementary Figure 11: R3C autocatalytic ligase tetrameric substrate complex. 14

Supplementary Figure 12: Bulk serial transfer comparing Cy5-A and Hyper-A substrates. 15

Supplementary Figure 13: *in vitro* characterization of F1 and Hyper-A under desired buffer conditions and optimization of Hyper-A input concentrations 16

Supplementary Figure 14: Gel images of R3C ligase ribozyme F and the active substrate Hyper-A *in vitro*. 17

Supplementary Figure 15: Activity of F1 ribozyme in the presence of GUVs in bulk solution. 18

Supplementary Figure 16: Gel image showing the activity of the encapsulated R3C autocatalytic system after a varying number of freeze-thaw cycles. 19

Supplementary Figure 17: Effect of freeze-thaw cycling on GUV diameter. 20

Supplementary Figure 18: Freeze-thaw cycling autocatalytic replicator in bulk solution. 21

Supplementary Figure 19: Representative gel image showing the activity of the encapsulated R3C system following serial dilutions in substrate encapsulated GUV feedstock 22

Supplementary Figure 20: PAGE analysis for RNA species identification. 23

Supplementary Methods 24

Supplementary References 26

Materials

1-palmitoyl-2-oleoyl-glycero-3-phosphocholine (POPC) was purchased from Avanti Polar. Atto 647N-functionalized DOPE (1,2-dioleoyl-sn-glycero-3-phosphoethanolamine) lipids were acquired from AttoTEC GmbH (Siegen, Germany) dissolved in chloroform at 1 mg/ml. For fluorescence detection inside GUVs, AlexaFluor568 NHS ester (Thermo Fischer Scientifics, Waltham, MA, USA) and HPLC-purified 5'-Atto488-(CA)13-3' and 5'-Atto655-(CA)13-3' oligonucleotides (both IDT, Coralville, IA, USA) were purchased. SensoPlate glass bottom microtiter plates (384 wells, V_{well} = 130 µL) were obtained from Greiner Bio-One and aluminium-coated sealing film ROTALIBO® was purchased from Carl Roth. All glass surfaces were passivated using a 10 mg/ml Pluronic F-127 (Sigma-Aldrich) solution according to the manufacturer's instructions. Mineral oil, dimethyl sulfoxide (DMSO), Rotiphorese 40% (19:1 acrylamide:bis-acrylamide) ready-to-use solution, TEMED, chloroform, *N*-(2-Hydroxyethyl)piperazine-*N'*-(3-propanesulfonic acid) (EPPS), ethylenediaminetetraacetic acid (EDTA), sodium acetate (NaOAc), Triton X-100, bromophenol blue, ammonium persulfate (APS) and urea were all purchased from Carl Roth. Formamide, 5-Carboxy-tetramethylrhodamine *N*-succinimidyl ester (TAMRA-NHS-ester), polyethylene glycol 8000 (PEG8000), absolute ethanol, 2-propanol (isopropanol), D-glucose, boric acid, glacial acetic acid, sodium tetraborate hexahydrate (NaB) were purchased from Sigma. Magnesium chloride ($MgCl_2$) hexahydrate and tris(hydroxymethyl)aminomethane (Tris) were purchased from Th.Geyer. D-sucrose, dithiothreitol (DTT), spermidine and RNA-grade glycogen were purchased from Thermo Fisher. Gamma-aminohexyl-GTP and NTPs were purchased from Jena Bioscience. Sodium hydroxide pellets and 37% hydrochloric acid solution were purchased from VWR. *E. coli* inorganic pyrophosphatase (IPP), T4 RNA ligase 2 (T4Rnl2) and the Monarch RNA cleanup kit (50µg) were purchased from NEB. Pre-coated TLC plates were purchased from Machery-Nagel. DNA oligonucleotides were synthesized by IDT. RNA was either transcribed in-house or purchased from IDT when mentioned. Cellulose acetate filter spin columns (Spin-X) were bought from Costar. Ultrapure Milli-Q water (Millipore) was used for all experiments in this study.

Supplementary Tables

Supplementary Table 1. Table of sequences used in this study.

Name	Length	5' end	Sequence (5' to 3')	3' end	Source	Reference
HH-min	39	-	GGGAGCUGAACUGAU GAGUCCGU GAGGACGAAAGGCAC A	-	IVT	Drobot et al. 2018
HH-mut*	39	-	GGGAGCUGAACU**A**AU GAGUCCGU GAGGAC**A**AAAGGCAC A	-	IVT	Drobot et al. 2018
HH-FQ-Sub	12	FAM	UGCCUCUUCAGC	BHQ1	IDT	Drobot et al. 2018
HH-Sub	27	Cy5	UUCACCUCAGAACUG UGCCUCUUCAGC	-	IDT	- this paper -
R3C ligase F1	66	-	GGAAGUUGUUAUCAC UUGUUACGUAAGUAA CAC UUUGGAUGGGUUGAA GUAUGAGACCGCAAC UUA	-	IVT	Robertson et al. 2014
Hyper-A	52	-	GGAAGUUGUUAUCAC UUGUUACGUAAGUAA CAC UUUGGAUGGGUUGAA GUAU	-	IDT	Robertson et al. 2014
Cy5-A*	52	Cy5	GGAAGUUGUUAUCAC UUGUUACGUAAGUAA CAC UUUG**A**AUGGGUUGAA GUAU	-	IDT	- this paper -
B	14	TP**	GAGACCGCAACUUA	-	IVT	Robertson et al. 2014
rt-F	66	-	GGAAGUUGUGUCUUA AUGUUACGUAAGUAA CAG AUGGAAUUGGUUGAA GUAUGAGACCGCAAC UUA	-	IVT	Olea et al. 2016
rt-A-short	10	-	GGUCUCAUAU	-	IDT	Olea et al. 2016
FQ-B	19	TAMRA-C6-TP**	GAGACCGCAACUUAC ACAU	BHQ2	IVT + Ligation	Olea et al. 2016
Penta-BHQ2	5	P**	CACAU	BHQ2	IDT	- this paper -

* Bases in bold red color are substitution mutations that render the sequences catalytically inactive.
** P = phosphate, TP = triphosphate

Supplementary Figures

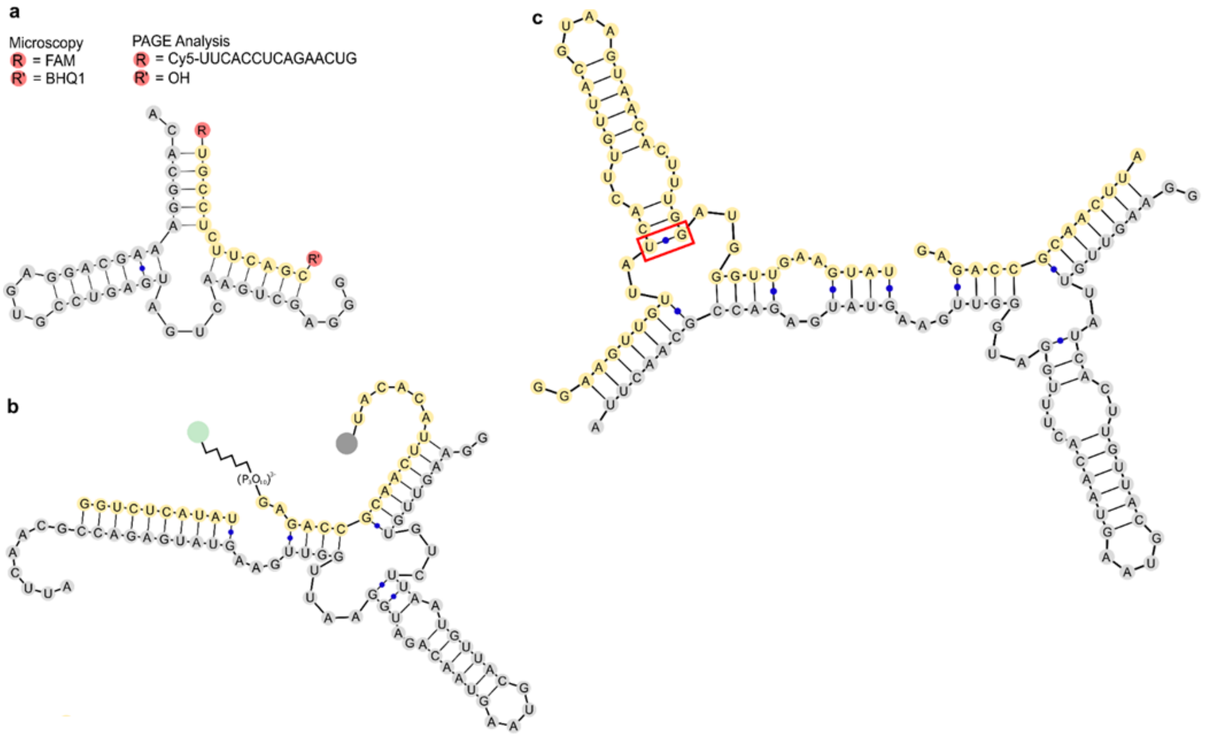

Supplementary Figure 1: Secondary structures of RNA systems used in the study.

a) The hammerhead ribozyme system shows the ribozyme in grey and the substrate in yellow. The substrate used for microscopy had a fluorophore and quencher attached to the 5' and 3' ends, respectively[1]. The substrate for PAGE analysis instead only had a nucleotide linker region and fluorophore at the 5' end. **b)** Structure of the R3C derived ligase (grey) and its modified substrate (yellow), adopted from Olea and Joyce[2]. Ligation results in the release of the fluorophore (green) and consequent increase in fluorescence. **c)** Structure of the R3C autocatalytic ligase F1 (grey) bound to its two substrates (yellow), from Robertson and Joyce[3]. The red box indicates the base pair that was mutated in this work from [U-G] to [U-A] in the Cy5-A version to downregulate background activity.

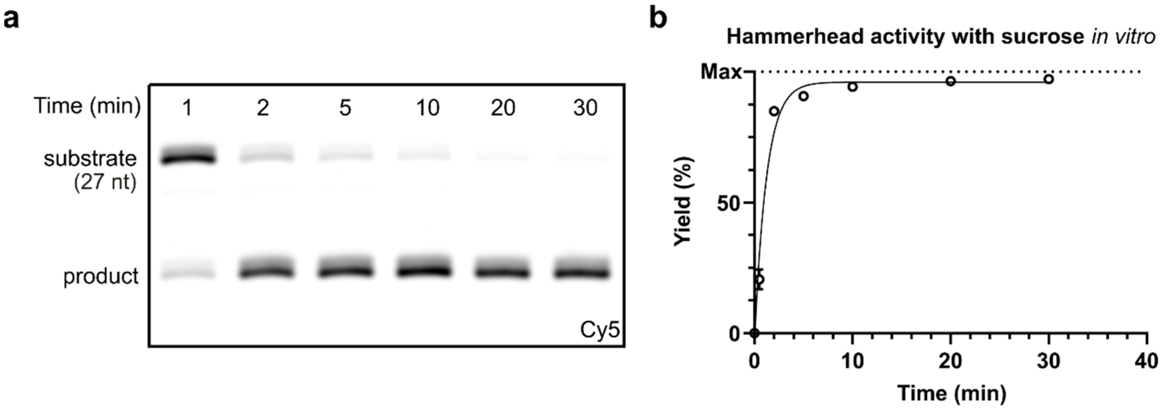

Supplementary Figure 2: Time course of hammerhead activity *in vitro* supplemented with sucrose.

a) Gel image showing time course of the cleavage of 5 µM HH-sub in the presence of 2.5 µM hammerhead ribozyme supplemented with 900 mM sucrose. **b)** Plot of HH-min cleavage yields from band intensity measurements of the gel image. Data points are fitted to an exponential plateau equation. Dot plots represent the mean values +/- SD from n = 3 independent experiments. The gel image is one of three independent experiments. See appendix for uncropped gel images.

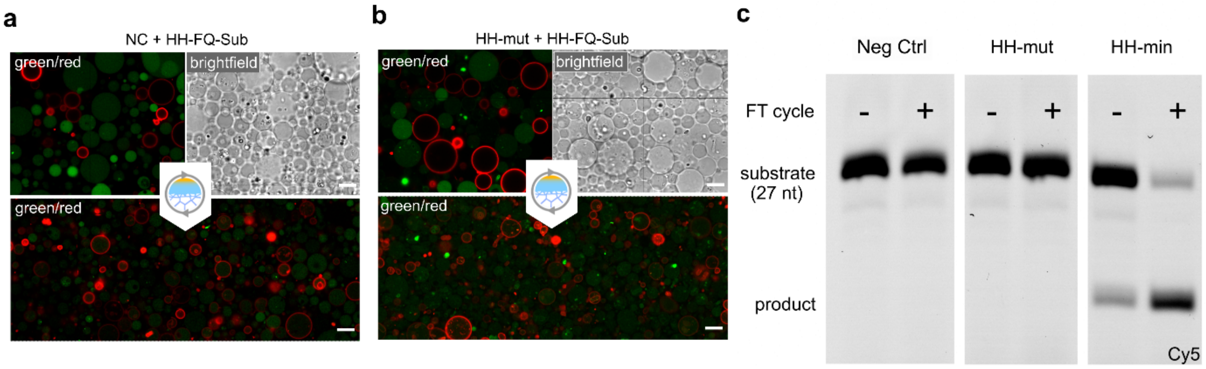

Supplementary Figure 3: Hammerhead activity in GUVs.

a-b) Control experiments of FT cycling on HH-sub cleavage with **a)** empty GUVs or **b)** the inactive HH-mut. The lack of fluorescence increase suggests that HH-FQ-sub is not cleaved by reaction conditions or FT-cycles. **c)** Denaturing PAGE analysis (20%) of encapsulated hammerhead cleavage assay pre- and post-cycling. Vesicles encapsulating 2.5 µM of Cy5-tagged substrate were incubated with empty vesicles (Neg Ctrl), vesicles containing 5 µM HH-mut or HH-min. All samples were analysed before and after a freeze-thaw (FT) cycle. All experiments were repeated 3 times with similar results. All scale bars represent 10 µm. The gel images are one of three independent experiments. See appendix for uncropped gel images.

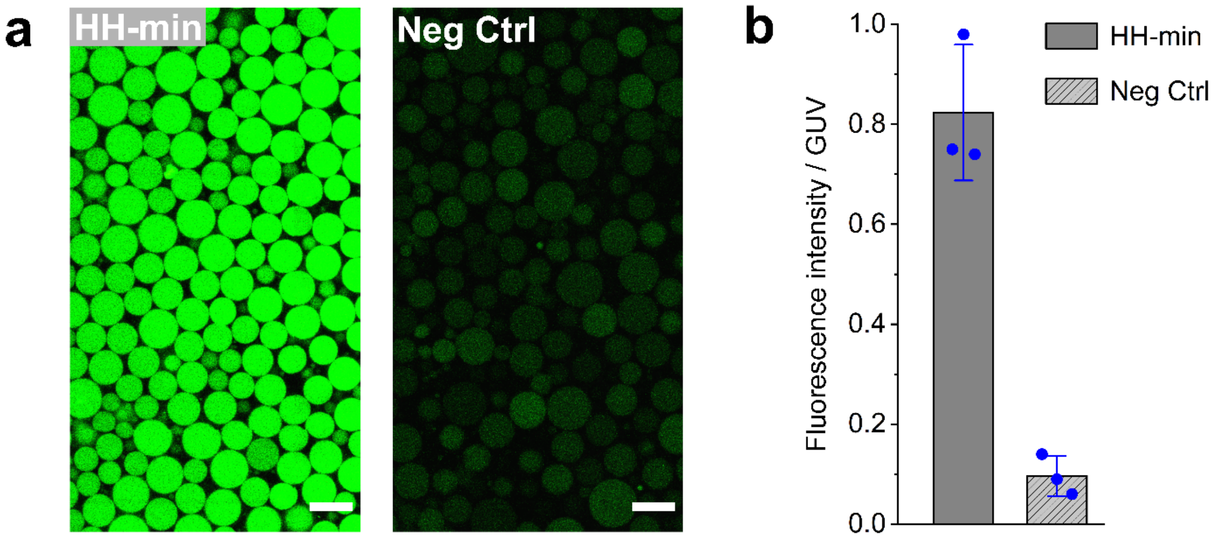

Supplementary Figure 4: Hammerhead activity in GUVs in the absence of freeze-thaw cycling.

a) GUVs encapsulating 5 µM HH-min and 2.5 µM HH-FQ-Sub (left) or 2.5 µM HH-FQ-Sub alone (right, Neg Ctrl). **b)** Normalized fluorescence intensities per GUV for the experiments shown in **a)**. Images were taken 30 min after GUV preparation at room temperature. An 8.3-fold increase in fluorescence intensity was observed when HH-min and HH-FQ-Sub were initially encapsulated in the same GUVs. Assuming that represents full cleavage (100%), this value could be used to estimate the percentage of substrate cleaved in the freeze-thaw experiment in Figure 1, i.e. the 6.7-fold increase corresponds to ~80% substrate cleavage. Data are presented as bar charts with mean values of GUV fluorescence +/- SD from n = 3 independent experiments. Scale bars represent 20 µm.

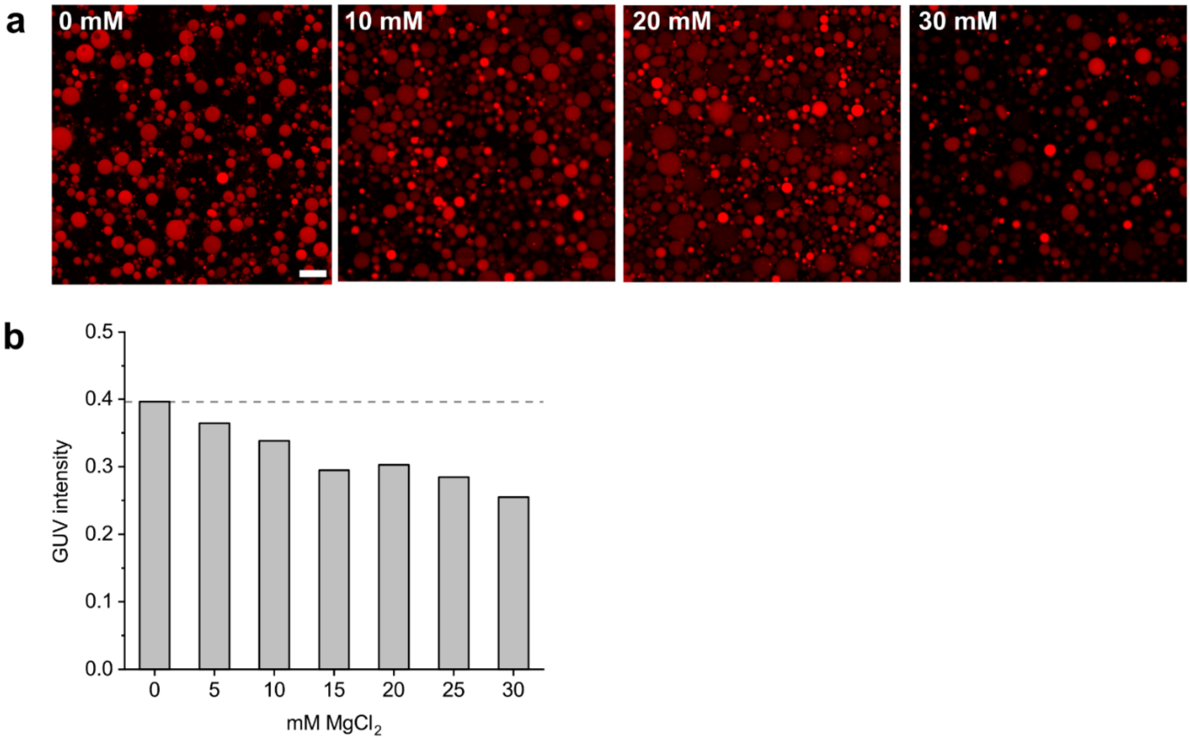

Supplementary Figure 5: Effect of $MgCl_2$ on emulsion transfer GUV preparation.

a) Confocal microscopy images of GUVs encapsulating a fluorescent marker (3 µM 5'-Atto655-(CA)13-3') prepared with increasing magnesium in the R3C buffers. **b)** Mean fluorescence intensities of GUVs extracted from the confocal images showing a decrease in mean GUV fluorescence intensity (suggestive of enhanced leakage) accompanied by an increase in encapsulation heterogeneity with increasing magnesium concentration. Although the effect of increasing magnesium concentration was suspected from observations made in other experiments presented in this paper, it was only quantified once here. Scale bar represents 20 µm.

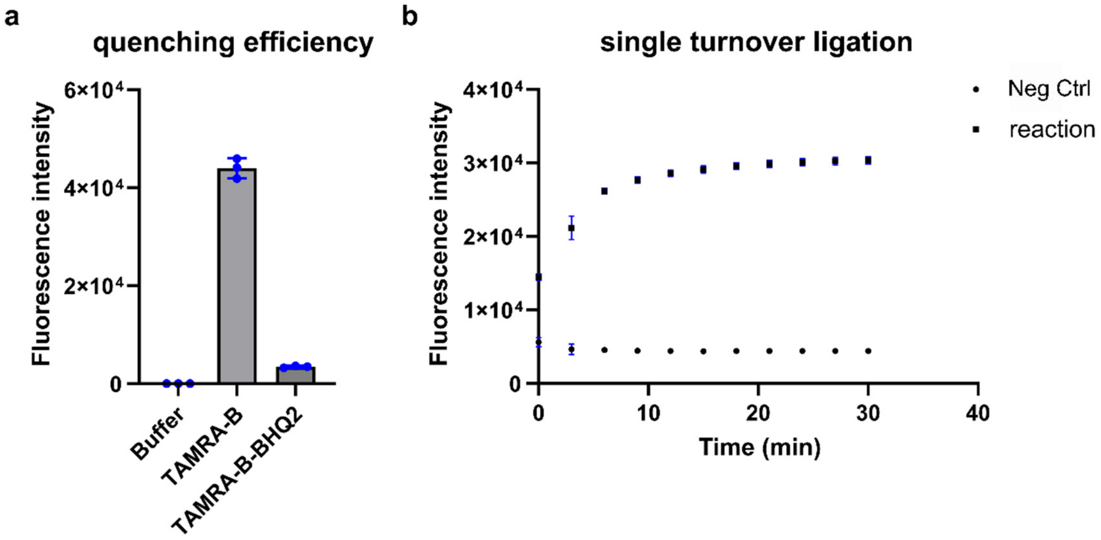

Supplementary Figure 6: *In vitro* characterization of the enzymatically synthesized TAMRA-B-BHQ2 (FQ-B) substrate.

a) Raw fluorescence of 1 µM FQ-B substrate before and after quencher attachment. Quenching efficiency was estimated to be 92%. **b)** Single turnover kinetics measuring fluorescence resulting from the ligation of 2 µM FQ-B and 5 µM rt-A-short by 5 µM rt-F. Negative control (Neg Ctrl) contains only substrate in buffer. The initial increase in fluorescence is not measured due to the lag time between transferring the samples to the microplate and starting the measurement in the microplate reader. Data are presented as mean values +/- SD from n = 3 independent experiments.

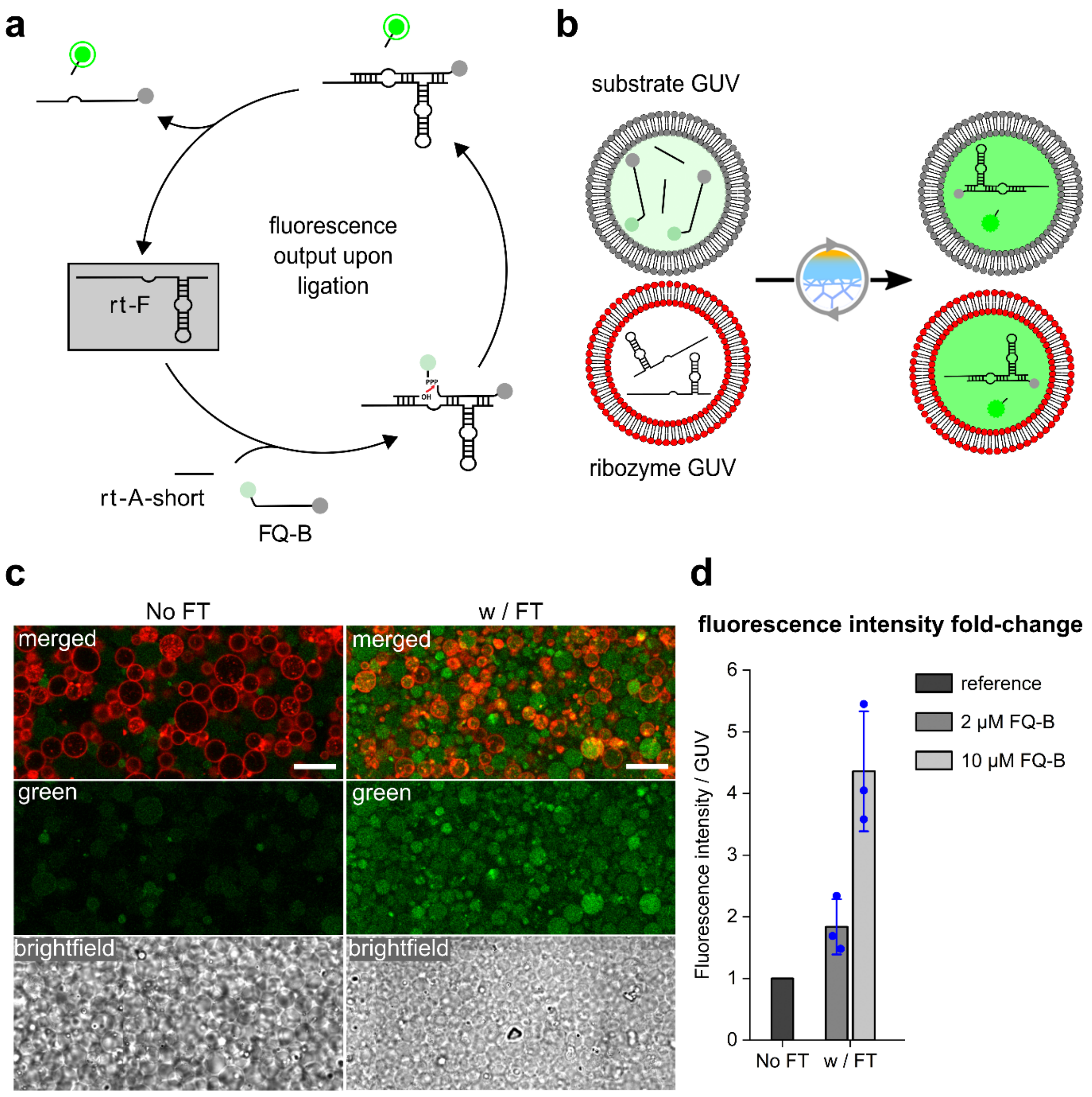

Supplementary Figure 7: Confocal microscopy analysis of freeze-thaw driven activity of a split R3C reporter ligase system.

a) Reaction cycle of the fluorescent R3C ligase reporter adopted from Olea and Joyce[2]. Here, the substrate FQ-B contains a 5(6)-carboxytetramethylrhodamine attached to the 5′-γ-phosphate and a 3’ BHQ2 quencher (for preparation see Supplemental Materials). **b)** Reaction setup in vesicles: the RNAs were separately encapsulated, GUV populations were combined in a 1:1 ratio before subjecting them to a freeze-thaw (FT) cycle. **c)** Confocal laser scanning microscopy images of the vesicle populations before and after a freeze-thaw cycle and short incubation time. **d)** Fold-change of fluorescence intensity per GUV after FT, normalised to the average fluorescence intensity of substrate and ribozyme GUVs before FT. The reactions exhibited a 1.8- and 4.4-fold increase in fluorescence for 2 and 10 µM FQ-B, respectively. Data are presented as bar charts with mean values of GUV fluorescence +/- SD from n = 3 independent experiments. Scale bars represent 20 µm.

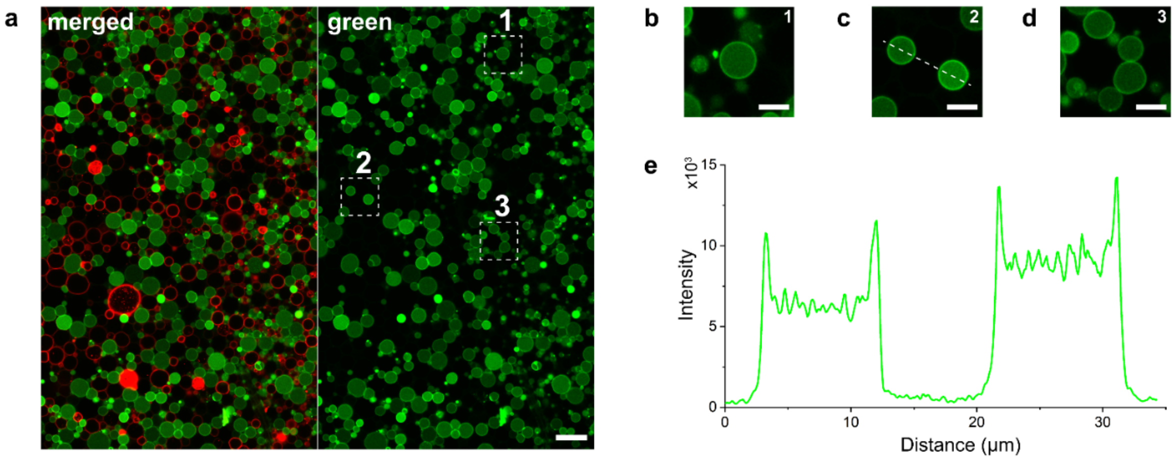

Supplementary Figure 8: Membrane interactions of encapsulated RNA.

a) Confocal microscopy images of the R3C ligase assay in GUVs before FT. Substrate vesicles (green) contained 10 μM FQ-B and 20 μM rt-A-short. GUV preparation under these conditions was performed at least 3 times. **b-d)** Close-up of boxes in **a)**. **e)** Fluorescence intensity across 2 GUVs along the dotted line shown in **c)**. The peaks at the extremities suggest that the fluorescent RNA (FQ-B) is interacting with the inner leaflet of the membrane thus promoting a local increase in concentration. Scale bars represent 30 μm in **a)** and 10 μm in **b-d)**. The short sequence of the substrate (19 nucleotides) and paucity of guanine bases suggest that the interaction stems from the conjugated fluorophore and / or quencher.

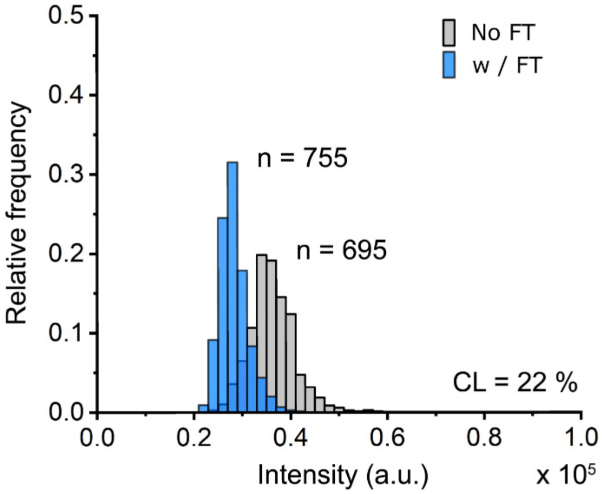

Supplementary Figure 9: Fluorescence intensity inside GUVs decreases upon freezing and thawing.

GUV intensity distributions before (grey) and after FT-cycling (blue). Vesicles initially contained 900 mM sucrose and 3 µM 5'-Atto655-(CA)$_{13}$-3'. GUV intensity distribution after freeze-thawing is shifted to lower intensities, suggesting that content loss (CL) occurs due to content dilution in the outer phase post-thawing. The number (n) represents the number of GUVs evaluated.

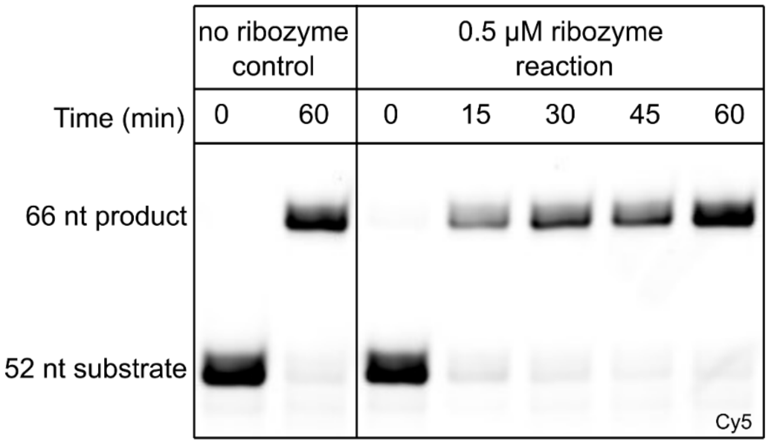

Supplementary Figure 10: Gel electrophoresis of RNA ligation by R3C ligase ribozyme F1 *in vitro*.

An initial test to assay the activity of the autocatalytic R3C ligase *in vitro* under the conditions desired that are compatible with vesicle production. The substrate concentrations (5 µM each) were 10-fold higher than ribozyme concentration (0.5 µM), and the A substrate contained 10% Cy5-tagged substrate. The control reaction shows a band at the final time point corresponding to product formation, suggesting residual activity in substrate oligonucleotides that can catalyse the ligation reaction in absence of ribozyme. The gel image is one of three independent experiments. See appendix for uncropped gel images.

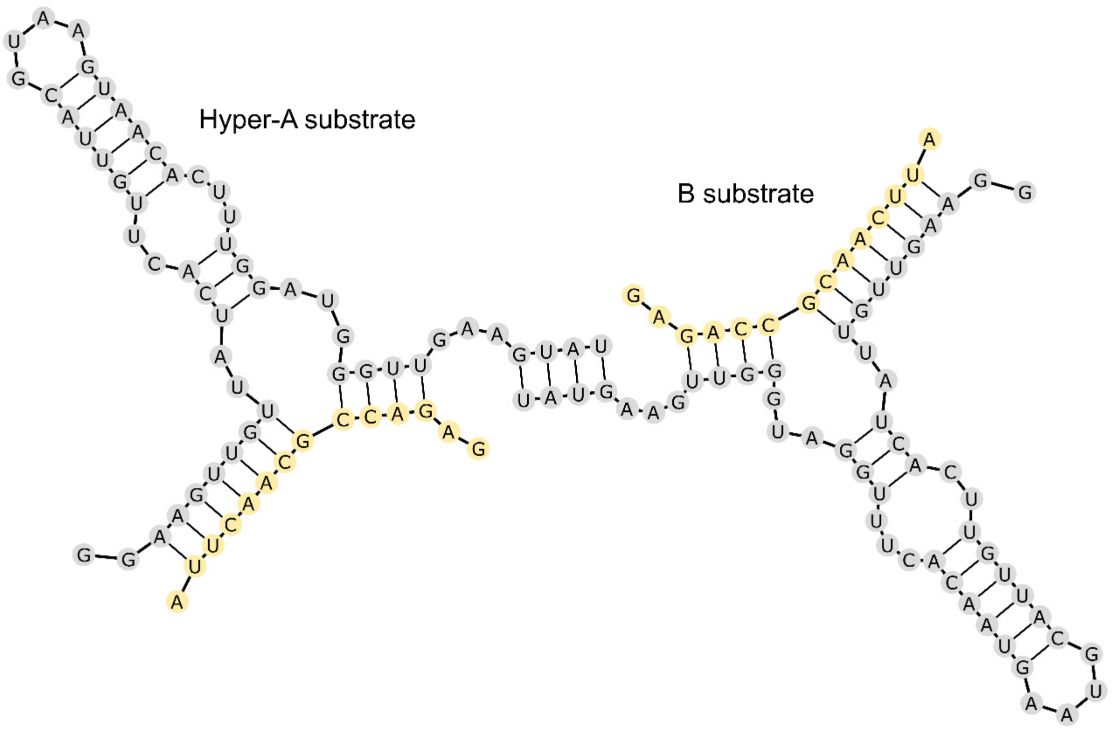

Supplementary Figure 11: R3C autocatalytic ligase tetrameric substrate complex.

Secondary structure of the 2A2B substrate tetramer that can form by complementary base pairing.

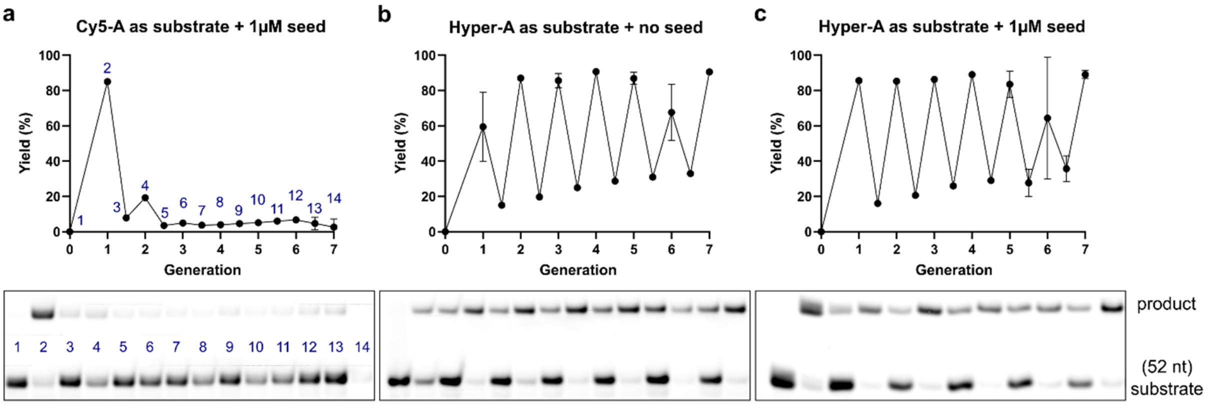

Supplementary Figure 12: Bulk serial transfer comparing Cy5-A and Hyper-A substrates.

Gel images and band intensity graphs showing the yield of newly synthesized ribozyme following serial transfers of the previous generations in unencapsulated bulk reactions. **(a)** The serial transfer of the ligation reaction of 10 μM Cy5-A and 10 μM B by 1 μM F1 shows that when the seeded ribozyme gets depleted the newly formed (Cy5-A + B) ribozyme is inactive. The serial transfer achieves a 10-fold dilution at every generation, totalling to a $1x10^6$ dilution of the starting reactants. **(b)** The serial transfer of the reaction between 10 μM Hyper-A (10% Cy5-A) and 10 μM B demonstrates the residual activity of Hyper-A in solution as opposed to Cy5-A, as well as the activity of the ligation product as a catalyst. **(c)** Positive control showing all reaction components together in solution. Dot plots are presented as mean values +/- SD from n = 3 independent experiments. The gel images are one of three independent experiments. See appendix for uncropped gel images.

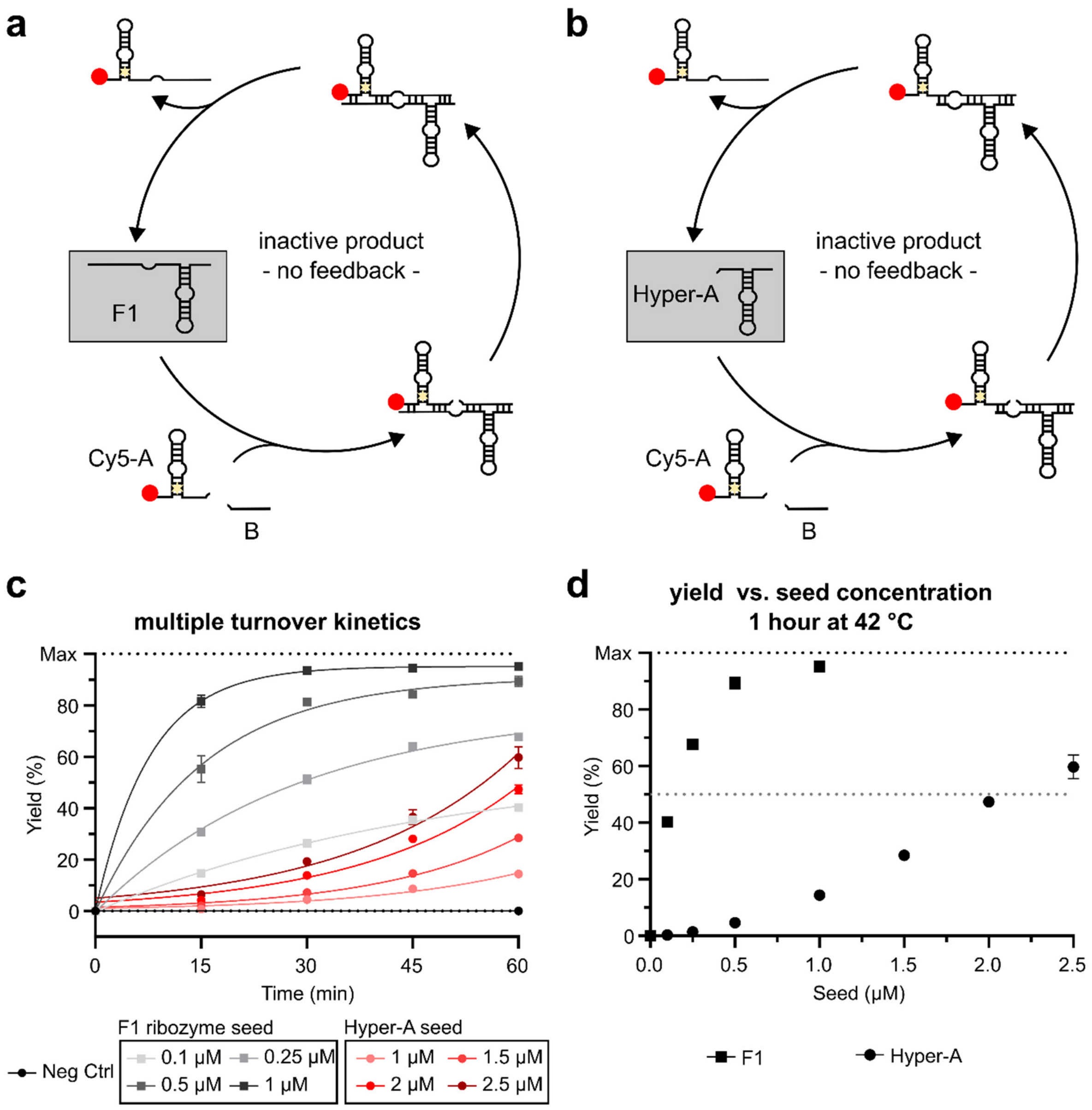

Supplementary Figure 13: *in vitro* characterization of F1 and Hyper-A under desired buffer conditions and optimization of Hyper-A input concentrations

Reaction cycles illustrating the ligation of 5 µM inactive Cy5-A substrate to 7.5 µM B substrate by **a)** F1 ribozyme or **b)** Hyper-A substrate. **c)** Plot showing the ligation yields of different seeded concentrations for F1 or Hyper-A. Gel images are shown in Supplementary Figure 14. Trendlines are for descriptive are shown for descriptive purposes only. **d)** Plot showing the ligation yields of F1 and Hyper-A as a function of initial seed concentration. At Hyper-A concentrations below or equal to 1 µM and in the absence of any initial full-length F1 only ~12% of ligation product formed after 60 min of incubation. In contrast, the addition of 1 µM "seed" F1 ribozyme led to an almost complete ligation (~90%) of Cy5-A and B already after 30 min. Dot plots are presented as mean values +/- SD from n = 3 independent experiments.

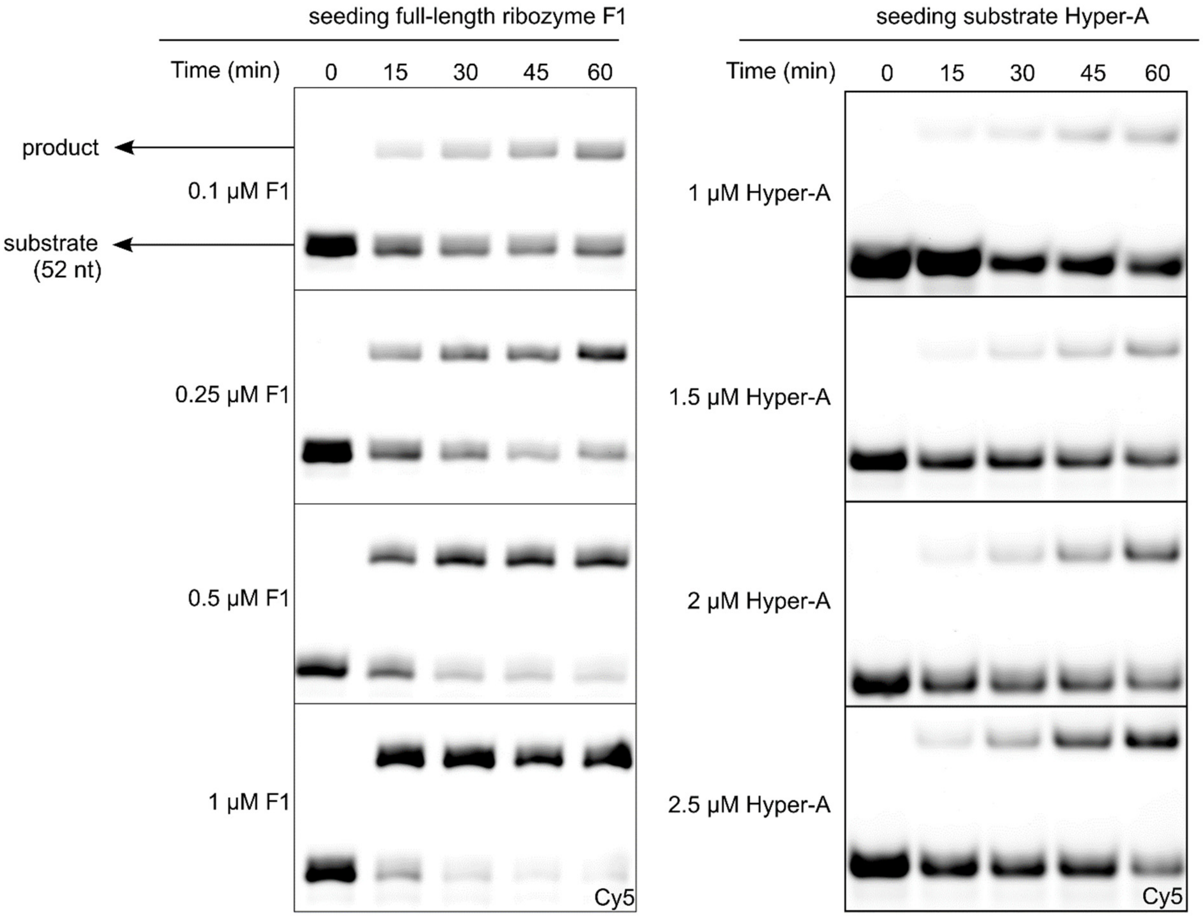

Supplementary Figure 14: Gel images of R3C ligase ribozyme F and the active substrate Hyper-A *in vitro*.

The ribozyme F1 and the Hyper-A substrate were seeded at different concentrations with 5 µM Cy5-A and 7.5 µM B substrate in 900 mM sucrose, 20 mM magnesium chloride and 50 mM EPPS at pH 8.5. Samples were incubated at 42 °C, quenched at the indicated times with 9 volumes of R3C loading buffer (25 mM EDTA in formamide, 0.01% bromophenol blue) and heat denatured at 85 °C for 5 minutes before analysis by denaturing PAGE (12%). The gel images are one of three independent experiments. See appendix for uncropped gel images.

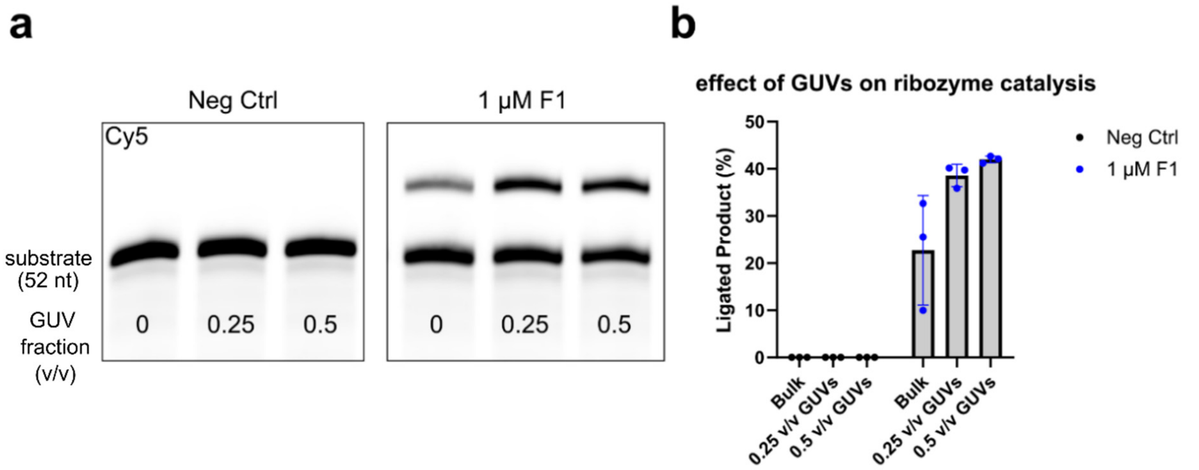

Supplementary Figure 15: Activity of F1 ribozyme in the presence of GUVs in bulk solution.

a) Representative PAGE gel image showing the negative control (0.5 μM Cy5-A; 1 μM B) without any ribozyme on the left and the reaction with 1 μM ribozyme on the right, at different fractions of GUVs present in solution. **b)** Graph showing band intensity integrations for the ligation reaction. The increase in ligation efficiency suggests that low concentration of organized lipid membranes offer a catalytic rate increase to the ribozyme in solution. Data are presented as box charts with mean values +/- SD from n = 3 independent experiments; individual data points are shown as dots. The gel images are one of three independent experiments. See appendix for uncropped gel images.

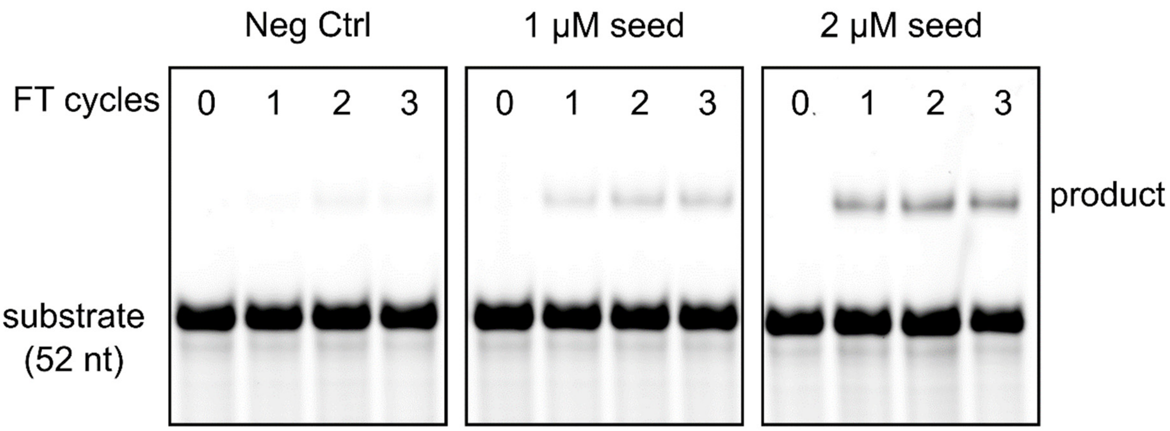

Supplementary Figure 16: Gel image showing the activity of the encapsulated R3C autocatalytic system after a varying number of freeze-thaw cycles.

Substrate GUVs were mixed with empty GUVs (Neg Ctrl) or with GUVs encapsulating 1 µM or 2 µM F1 ribozyme and were subjected to cycles of freezing and thawing followed by a 60-minute incubation period at 42 °C. RNA was recovered and quenched with GUV loading buffer, heat denatured and electrophoresed on a 12% polyacrylamide gel. To account for content loss and standardise band intensity, 20% more volume was loaded into the well for every additional freeze-thaw cycle applied to the sample. The gel images are one of three independent experiments. See appendix for uncropped gel images.

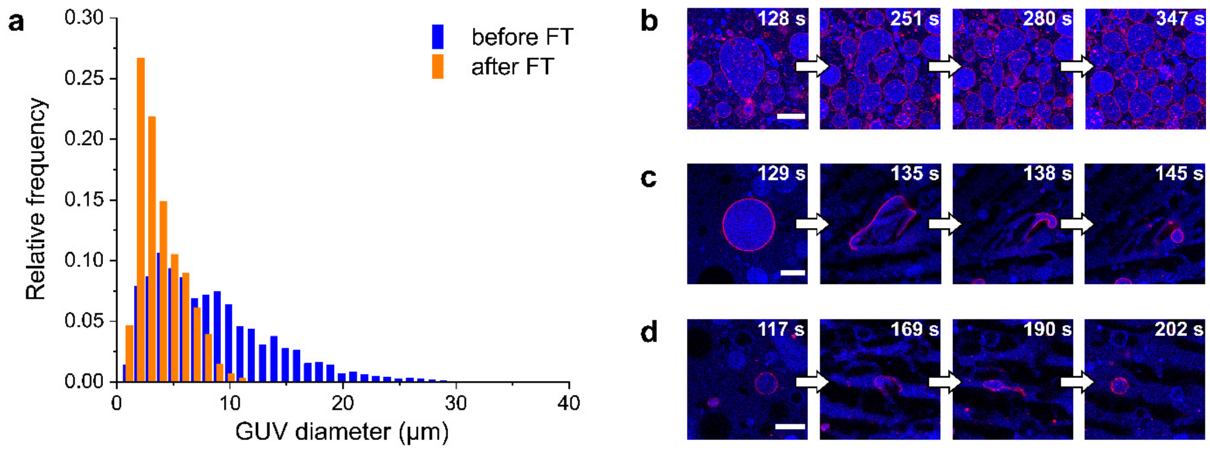

Supplementary Figure 17: Effect of freeze-thaw cycling on GUV diameter.

a) Relative frequency plot of GUV count per GUV diameter before and after freeze-thaw (FT). **b-d)** The decrease in GUV diameter as a result of FT can be explained by **b)** local changes in osmolarity, **c)** ice crystal formation and **d)** passage of GUVs through tight channels formed by frozen water. GUVs contained 900 mM sucrose and 3 µM Alexa Fluor 568 NHS ester. The surrounding phase consisted of a 900 mM glucose solution. Red colour corresponds to Atto 647N-DOPE. Individual movies of **b-d)** can be found as supplementary videos (Supplementary Movies 1-3, respectively). All scale bars represent 10 µm.

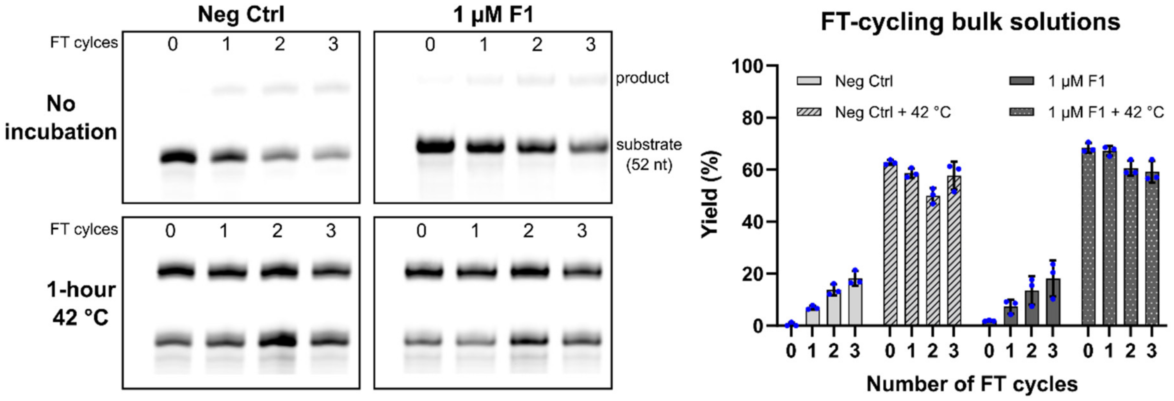

Supplementary Figure 18: Freeze-thaw cycling autocatalytic replicator in bulk solution.

Gel images and quantifications of yields from reactions containing 10 µM A (10% Cy5-A) and 15 µM B substrate, varying number of freeze-thaw cycles, with and without an incubation time at 42 °C. Samples that were not incubated at 42 °C show only background activity, attributed to slow catalysis at room temperature. In contrast, samples that were incubated at 42 °C showed substantial yields, irrespective of whether they were seeded with F1 ribozyme or not, due to background activity of Hyper-A in solution. Data are presented as box charts with mean values +/- SD from n = 3 independent experiments; individual data points are shown as dots. The gel images are one of three independent experiments. See appendix for uncropped gel images.

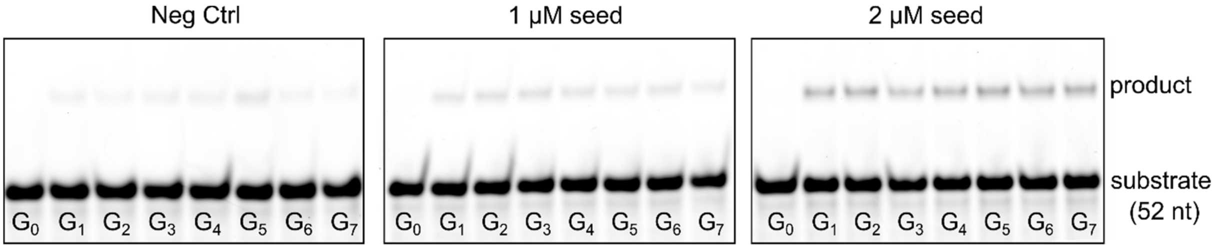

Supplementary Figure 19: Representative gel image showing the activity of the encapsulated R3C system following serial dilutions in substrate encapsulated GUV feedstock.

Substrate and ribozyme GUVs were prepared and combined, subjected to a freeze-thaw cycle and incubated for 60 minutes at 42 °C. Then, half the volume was sampled and the other half was transferred to an equal volume of fresh substrate feedstock GUVs and the cycle was repeated. Collected samples were washed with outer phase buffer, concentrated and suspended in 1-2 volumes GUV loading buffer (25 mM EDTA, 0.3% Triton X-100, 0.01% bromophenol blue in formamide). Samples were heat denatured at 85 °C for 5 minutes before denaturing PAGE analysis (12%). The gel images are one of three independent experiments. See appendix for uncropped gel images.

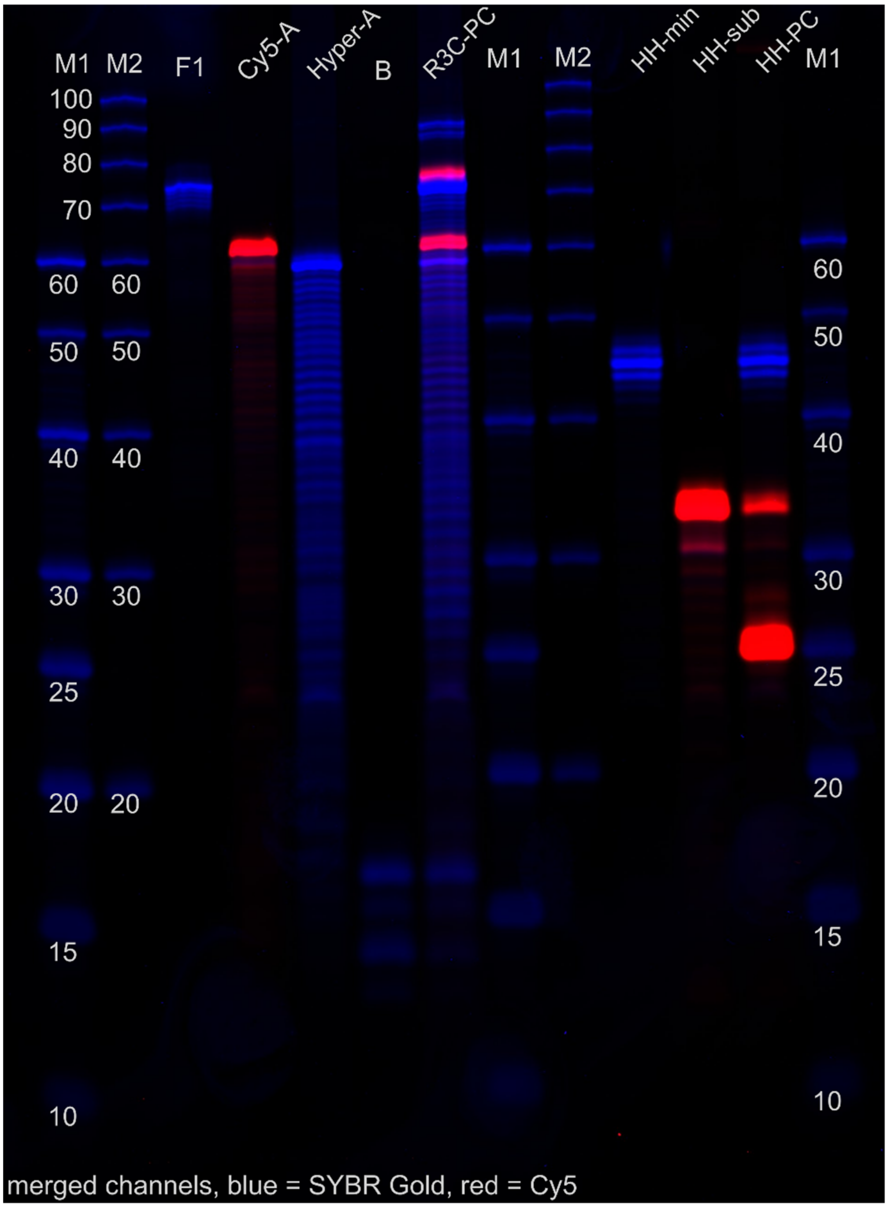

Supplementary Figure 20: PAGE analysis for RNA species identification.

Identification runs were performed to localize and verify the functionality of the different RNA species that were used here for analysis by PAGE. The R3C components are shown on the left-hand side: F1 ribozyme (1 µM), Cy5-A (1 µM), Hyper-A (10 µM) and B (15 µM) substrates, followed by a positive control lane (R3C-PC) in which the RNAs were allowed to react in R3C buffer (50 mM EPPS pH 8.5, 20 mM $MgCl_2$) at 42 °C for 30 minutes. The right-hand side shows Hammerhead system components: HH-min (5 µM), HH-sub (2.5 µM) followed by a positive control lane (HH-PC) in which the the RNAs were allowed to react in HH buffer (20 mM Tris-HCl pH 8.3, 4 mM $MgCl_2$) at 37 °C for 30 minutes. Samples were quenched with 4 volumes of the respective loading buffers, heat denatured and resolved on 15% denaturing PAGE. Following electrophoresis, the gel was stained with SYBR Gold and imaged in both SYBR (520 nm excitation) and Cy5 (658 nm excitation) channels. Marker lanes (M1 and M2) are the 10/60 and 20/100 ssDNA oligo ladders purchased from IDT. Bands below 20 nucleotides appear fainter due to poor binding of the dye, whereas the offset in molecular weight stems from the higher molecular weight of RNA monomers as opposed to DNA monomers (additional 2' OH) and, when applicable, from the attachment of the Cy5 dye for labelled substrates.

Supplementary Methods

(S2) Hammerhead assay in solution. Reactions were carried out in volumes of 50 µL. Substrate cleavage was monitored in a reaction containing 5 µM hammerhead ribozyme, 2.5 µM Cy5-tagged substrate in hammerhead buffer that consisted of 20 mM Tris-HCl pH 8.3, 900 mM sucrose, and 4 mM $MgCl_2$. The reaction was set up on ice and started by the addition of $MgCl_2$ and incubating at 25 °C. Samples were taken at specific time points by quenching 5 µL of reaction with 20µL of hammerhead loading buffer containing 10 mM EDTA, 0.01% bromophenol blue, 98% formamide and placed on ice. After all samples were collected, they were denatured at 85 °C for 5 minutes and cooled quickly on ice before denaturing PAGE analysis (20%). Gel imaging and analysis were performed as described in the main methods. Data was fitted to an exponential plateau function on GraphPad Prism.

(S6) Determination of quenching efficiency and single turnover kinetics for FQ-B. To determine the quenching efficiency, fluorescence measurements for 1 µM of either TAMRA-B or TAMRA-B-BHQ2 (FQ-B) in R3C buffer containing 20 mM magnesium chloride, 50 mM EPPS pH 8.5, 900 mM sucrose were acquired in a microplate reader by exciting at 545 nm and detecting emission at 590 nm. The quenching efficiency was estimated using the equation QE = (1-(Fluorescence [TAMRA-B-BHQ2] / Fluorescence [TAMRA-B])) * 100. For single turnover kinetics, reactions were assembled on ice then transferred to a microplate and fluorescence (545 / 590 nm) was measured over time every 120 seconds in a microplate reader preheated to 37 °C. The reaction contained 2 µM FQ-B with 5 µM of each rt-F and rt-short-A in a buffer composed of 20 mM magnesium chloride, 50 mM EPPS pH 8.5, 900 mM sucrose, while the control had only the fluorescent substrate in buffer.

(S7) Synthesis of FQ-B Substrate. TAMRA-NHS-Ester was bought from Sigma. gamma-aminohexyl-GTP was bought from Jena Bioscience. A 5'-phosphorylated pentamer with a 3'-end quencher (BHQ2) was ordered from IDT. The substrate was first *in vitro* transcribed with the modified initiator nucleotide. The reaction differed from the IVT described above by having 6 mM ATP, UTP and CTP, 2 mM GTP, and 4 mM gamma-aminohexyl-GTP. The resulting RNA was column purified (Monarch RNA Clean-up Kit) and reacted with the TAMRA-NHS-ester according to manufacturer's instructions. Briefly, around 60 ODs of the 5' aminohexyl RNA was dried under vacuum and resuspended in 600 µL sodium tetraborate pH 8.4 then combined with 100 µL of TAMRA-NHS-ester in DMSO at 10 µg/µL of fluorophore and incubated for 4 hours in a dry block at 25 °C, 600 rpm. The RNA was precipitated in 1.2 volumes of isopropanol, washed once with 80% ethanol, resuspended in ultrapure water and quantified on a Nanodrop as described above. Subsequently, the fluorophore-tagged RNA was ligated to the 3' quencher-tagged pentamer with T4 RNA ligase 2 (NEB) and a DNA splint complementary to the 15 nucleotides from the 3'-end. The reaction setup (200 µL – 1 mL) was as follows: 3 µM of TAMRA-B, 3.5 µM Penta-BHQ2 (Supplementary Table 1), 3.5 µM DNA splint, 8% DMSO, 1X T4 RNA ligase 2 buffer, 9 mM $MgCl_2$, 100 µM ATP, 50 U T4 RNA ligase 2, 15% PEG8000. The nucleic acids were mixed with water and DMSO, heated to 85 °C and placed on ice for annealing. The remaining reagents were added in the order listed. The reaction was incubated at 25 °C for 1 hour, then at 16 °C for 16 hours. After isopropanol precipitation, the pellet was suspended in formamide and gel purified on a denaturing 20% PAGE gel. Recovery and quantification of RNA was as described above.

(S7) Fluorescent R3C ligase assay in GUVs for microscopy. GUVs encapsulating the R3C buffer (900 mM sucrose, 50 mM EPPS pH 8.5, 20 mM $MgCl_2$) were prepared as described in the main methods. One population was membrane-labelled and contained the ribozyme rt-F, the other contained the substrates rt-A-short and FQ-B. Ribozyme and substrates were encapsulated at varying concentrations in two experimental approaches. Concentrations were either 20/15/10 µM or 5/5/2 µM (for rt-F/rt-A-short/FQ-B, respectively). The outer phase consisted of 900 mM of glucose and R3C buffer. Freezing and thawing was implemented as described in the main methods. Images were taken at room temperature before and after freeze-thawing. Further images were taken after a subsequent 1 h incubation at 37 °C.

(S10) R3C ligase characterization in solution. Activity was assayed in 50 mM EPPS buffer pH 8.5 supplemented with 20 mM magnesium chloride, 900 mM sucrose at 42 °C. The substrate concentrations

were equal in both control and reaction: 0.5 µM Cy5-A, 4.5 µM Hyper-A, and 12 µM B, whereas the ribozyme was added at a final concentration of 0.5 µM in the reaction only. Samples were collected at the specified time points by quenching 1 µL in 9 µL R3C loading buffer (25 mM EDTA in formamide). Quenched samples were then heat denatured at 85 °C for 5 minutes and RNAs separated by 8 M urea 1X TBE denaturing PAGE (12%). The gel was transferred to the scanner and imaged in the Cy5 channel.

(S12) Serial transfer of R3C autocatalytic ligase in bulk solution. Typical 10 µL reaction volumes were prepared, the first containing 10 µM Cy5-A with 10 µM of B and 1 µM ribozyme in 20 mM $MgCl_2$ and 50 mM EPPS pH 8.5. The second containing 9 µM Hyper-A, 1 µM Cy5-A and 10 µM B without any ribozyme in the same buffer, and lastly a positive control with 9 µM Hyper-A, 1 µM Cy5-A, 10 µM B and 1 µM F1 ribozyme. The reactions were incubated for 30 minutes at 42 °C, after which 1 µL of the reaction was transferred to 9 µL of fresh substrate mix and the incubation was repeated. This was done several times to achieve $1x10^6$ dilution of the starting reactants. Samples were taken at the initial and final timepoints of a generation by quenching 1 µL of reaction with 9 µL R3C RNA loading buffer (25 mM EDTA in formamide). Samples were heat denatured at 85 °C for 5 minutes before denaturing PAGE analysis (12%).

(S13) R3C autocatalytic ligase assay in bulk solution. To determine the activity of the R3C ligase ribozyme in vitro, only the Cy5-tagged inactive A substrate was used as a reporter for ligation yields. 10 µL reactions were prepared on ice, with 5 µM Cy5-A, 7.5 µM B, and varying concentrations of either ribozyme F1 or the unmodified substrate Hyper-A. The R3C buffer consisted of 50 mM EPPS pH 8.5, 900 mM sucrose, and 20 mM $MgCl_2$. The reaction was started by the addition of $MgCl_2$ and incubating it at 42 °C. Samples were consistently taken at specific time points by quenching 1 µL of the reaction with 9 µL of R3C RNA loading buffer (containing 25 mM EDTA, 95% formamide and 0.01% bromophenol blue) and placed on ice. After all samples were collected, they were denatured at 85 °C for 5 minutes and immediately placed on ice, before denaturing urea-PAGE analysis (12%). Gel imaging and analysis were performed as described in the main methods. The data points of the ribozyme F1 were fitted to an exponential plateau function while the data points of the Hyper-A substrate were fitted to an exponential growth function on GraphPad Prism.

(S15) Activity of autocatalytic R3C ligase in the presence of GUVs. Empty GUVs were prepared according to the methods section with R3C buffer (20 mM $MgCl_2$ and 50 mM EPPS pH 8.5). A 10 µL reaction volume containing R3C buffer and either 0.5 µM Cy5-A and 1 µM B only (Neg Ctrl) or the same substrate concentrations with 1 µM F1 ribozyme. The reactions were prepared on ice, then increasing volume fractions of GUVs were added to the reaction (0 v/v = 10 µL of 1X buffer was added; 0.25 v/v = 5 µL 1x buffer and 5 µL GUV preparation was added; 0.5 v/v = 10 µL GUV preparation was added). The reactions were then incubated for 1 hour at 42 °C and stopped with 30 µL GUV loading buffer. After heat denaturation at 85 °C for 5 minutes, samples were analysed by denaturing urea-PAGE (12%) and imaged as described previously.

(S18) Activity of autocatalytic R3C ligase in bulk solution under freeze-thaw conditions. In a total reaction volume of 10 µL, the different components (9 µM Hyper-A; 1 µM Cy5-A; 15 µM B) were combined at room temperature either with 1 µM F1 (grey bars) or without any F1 ribozyme seed (Neg Ctrl, black bars) in 1X R3C buffer (50 mM EPPS; 20 mM $MgCl_2$) and subjected to several freeze-thaw cycles (one FT cycle comprises 10 minutes at -80 °C and 10 minutes at room temperature). After each cycle, 1 µL of sample was quenched with 9 µL RNA loading buffer (10 mM EDTA, 98% formamide, 0.05% bromophenol blue) for analysis. When mentioned, the reactions were further incubated for 60 minutes at 42 °C and sampled in the same way. The samples analysed by denaturing urea-PAGE (12%) after heat denaturation at 85 °C for 5 minutes.

Supplementary References

1. Drobot B, Iglesias-Artola JM, le Vay K, et al. Compartmentalised RNA catalysis in membrane-free coacervate protocells. *Nat Commun*. 2018;9(1):3643. doi:10.1038/s41467-018-06072-w

2. Olea C, Joyce G. Real-Time Detection of a Self-Replicating RNA Enzyme. *Molecules*. 2016;21(10):1310. doi:10.3390/molecules21101310

3. Robertson MP, Joyce GF. Highly Efficient Self-Replicating RNA Enzymes. *Chem Biol*. 2014;21(2):238-245. doi:10.1016/j.chembiol.2013.12.004
