# Supplementary material for: Periodic temperature changes drive the proliferation of self-replicating RNAs in vesicle populations
Source: Nat Commun. 2023 Mar 3;14:1222. doi: 10.1038/s41467-023-36940-z (PMC9984477; doi:10.1038/s41467-023-36940-z)
Supplement: Supplementary file 3 — Description of Additional Supplementary Files [file 41467_2023_36940_MOESM3_ESM.pdf]

Description of Additional Supplementary Files

Supplementary Movie 1:
Freeze-thaw-induced changes in local osmolarity cause decrease in GUV diameter. GUVs contained 900 mM sucrose and 3 µM Alexa Fluor 568 NHS ester. The surrounding phase consisted of a 900 mM glucose solution. Red colour corresponds to Atto 647N-DOPE. The movie shows the moment of sample thawing after liquid nitrogen-induced freezing. Osmolarity-driven membrane instabilities results in the fragmentation of a giant vesicle into smaller vesicles. See Methods section for explicit details about the experimental setup used for imaging.

Supplementary Movie 2:
Effect of freeze-thaw-induced ice crystal formation on GUV diameter. GUVs contained 900 mM sucrose and 3 µM Alexa Fluor 568 NHS ester. The surrounding phase consisted of a 900 mM glucose solution. Red colour corresponds to Atto 647N-DOPE. The movie shows the moment of sample freezing shortly after pouring liquid nitrogen into the surrounding wells. The formation of ice crystals (dark, non-fluorescent background) provokes the rupture of GUVs, which, at times, recover into vesicles with smaller diameters. See Methods section for details about the experimental setup used for imaging.

Supplementary Movie 3:
The passage of GUVs through ice channels during the freeze-thaw cycling affects GUV diameter. GUVs contained 900 mM sucrose and 3 µM Alexa Fluor 568 NHS ester. The surrounding phase consisted of a 900 mM glucose solution. Red colour corresponds to Atto 647N-DOPE. The initial movie contained a number of low-quality images/frames between the timeframes 117 s and 153 s due to focussing issues. For this reason, the movie starts at 153 s and neither includes the first image of the shown image series ('117 s') nor the moment of sample freezing. The movie shows the moment of sample thawing, a third example of how GUVs lose membrane material during freeze-thaw cycles. Thin channels that form between ice are able to extrude vesicles resulting in a decrease in GUV diameter. See Methods section for details about the experimental setup used for imaging.
