# Supplementary material for: Periodic temperature changes drive the proliferation of self-replicating RNAs in vesicle populations
Source: Nat Commun. 2023 Mar 3;14:1222. doi: 10.1038/s41467-023-36940-z (PMC9984477; doi:10.1038/s41467-023-36940-z)
Supplement: Supplementary file 7 — Reporting Summary [file 41467_2023_36940_MOESM7_ESM.pdf]

nature portfolio

Corresponding author(s): Petra Schwille, Hannes Mutschler

Last updated by author(s): Feb 17, 2023

Reporting Summary

Nature Portfolio wishes to improve the reproducibility of the work that we publish. This form provides structure for consistency and transparency in reporting. For further information on Nature Portfolio policies, see our Editorial Policies and the Editorial Policy Checklist.

Statistics

For all statistical analyses, confirm that the following items are present in the figure legend, table legend, main text, or Methods section.

n/a	Confirmed	
☐	☒	The exact sample size (*n*) for each experimental group/condition, given as a discrete number and unit of measurement
☐	☒	A statement on whether measurements were taken from distinct samples or whether the same sample was measured repeatedly
☒	☐	The statistical test(s) used AND whether they are one- or two-sided<br>*Only common tests should be described solely by name; describe more complex techniques in the Methods section.*
☒	☐	A description of all covariates tested
☒	☐	A description of any assumptions or corrections, such as tests of normality and adjustment for multiple comparisons
☐	☒	A full description of the statistical parameters including central tendency (e.g. means) or other basic estimates (e.g. regression coefficient) AND variation (e.g. standard deviation) or associated estimates of uncertainty (e.g. confidence intervals)
☒	☐	For null hypothesis testing, the test statistic (e.g. *F*, *t*, *r*) with confidence intervals, effect sizes, degrees of freedom and *P* value noted<br>*Give P values as exact values whenever suitable.*
☒	☐	For Bayesian analysis, information on the choice of priors and Markov chain Monte Carlo settings
☒	☐	For hierarchical and complex designs, identification of the appropriate level for tests and full reporting of outcomes
☒	☐	Estimates of effect sizes (e.g. Cohen's *d*, Pearson's *r*), indicating how they were calculated

Our web collection on statistics for biologists contains articles on many of the points above.

Software and code

Policy information about availability of computer code

Data collection	For microscopy image acquisition, Zeiss Zen Black v13.0.2.518 was used.<br>Gel scans were acquired using the Azure Sapphire Capture Software v1.7.0319.0 and band integrations were performed on AzureSpot Pro v2.2.167
Data analysis	Image analysis was performed on Fiji v1.53c, while data processing and plotting was done on OriginPro 2020b, v9.7.5.184 and on GraphPad Prism 9.3.0

For manuscripts utilizing custom algorithms or software that are central to the research but not yet described in published literature, software must be made available to editors and reviewers. We strongly encourage code deposition in a community repository (e.g. GitHub). See the Nature Portfolio guidelines for submitting code & software for further information.

Data

Policy information about availability of data

All manuscripts must include a data availability statement. This statement should provide the following information, where applicable:

- Accession codes, unique identifiers, or web links for publicly available datasets
- A description of any restrictions on data availability
- For clinical datasets or third party data, please ensure that the statement adheres to our policy

Source data are provided with this paper. The source data for all charts and graphs are provided in an Excel document where each worksheet contains the relevant

data for each figure and supplementary figure. In addition, all uncropped gel scans used for the figures are provided on the respective worksheet in the Excel document.

Human research participants

Policy information about studies involving human research participants and Sex and Gender in Research.

Reporting on sex and gender	This data was not collected because this study does not involve human research participants.
Population characteristics	This data was not collected because this study does not involve human research participants.
Recruitment	This data was not collected because this study does not involve human research participants.
Ethics oversight	This data was not collected because this study does not involve human research participants.

Note that full information on the approval of the study protocol must also be provided in the manuscript.

Field-specific reporting

Please select the one below that is the best fit for your research. If you are not sure, read the appropriate sections before making your selection.

[x] Life sciences [ ] Behavioural & social sciences [ ] Ecological, evolutionary & environmental sciences

For a reference copy of the document with all sections, see nature.com/documents/nr-reporting-summary-flat.pdf

Life sciences study design

All studies must disclose on these points even when the disclosure is negative.

Sample size	No sample size calculation was performed. Sample sizes were chosen based on the minimum number of independent experiments that allows statistical analyses, in this case three.
Data exclusions	No data were excluded from the analyses.
Replication	Experiments were reliably reproduced three times using different batches of reagents and buffers, except were explicitly mentioned in the figure legends (Supplementary Figure 5).
Randomization	Not applicable to this study because group allocation was not carried out.
Blinding	Not applicable to this study because group allocation was not carried out.

Reporting for specific materials, systems and methods

We require information from authors about some types of materials, experimental systems and methods used in many studies. Here, indicate whether each material, system or method listed is relevant to your study. If you are not sure if a list item applies to your research, read the appropriate section before selecting a response.

Materials & experimental systems

n/a	Involved in the study	
[x]	[ ]	Antibodies
[x]	[ ]	Eukaryotic cell lines
[x]	[ ]	Palaeontology and archaeology
[x]	[ ]	Animals and other organisms
[x]	[ ]	Clinical data
[x]	[ ]	Dual use research of concern

Methods

n/a	Involved in the study	
[x]	[ ]	ChIP-seq
[x]	[ ]	Flow cytometry
[x]	[ ]	MRI-based neuroimaging
